# Thinking like a Lawyer—Human Rights and Their Association with the Plastic Surgeon of Today

**DOI:** 10.1007/s00266-022-02990-9

**Published:** 2022-08-03

**Authors:** Leonard Knoedler, Berkin Oezdemir, Philipp Moog, Lukas Prantl, P. Niclas Broer, Christoph Knoedler, Ulrich M. Rieger, Markus Perl, Sarah von Isenburg, Ulrich M. Gassner, Doha Obed, Valentin Haug, Adriana C. Panayi, Samuel Knoedler

**Affiliations:** 1 Department of Plastic, Hand and Reconstructive Surgery, University Hospital Regensburg, Regensburg, Germany; 2 Faculty of Medicine, University Hospital Heidelberg, Heidelberg, Germany; 3 Department of Plastic Surgery and Hand Surgery, Klinikum Rechts der Isar, Technical University of Munich, Munich, Germany; 4 Department of Plastic, Hand and Burn Surgery, Bogenhausen Academic Teaching Hospital, Munich, Germany; 5 Faculty of Applied Social and Health Sciences, Regensburg University of Applied Sciences, Regensburg, Germany; 6 Department of Plastic and Aesthetic, Reconstructive and Hand Surgery, AGAPLESION Markus Hospital, Academic Teaching Hospital of the J.W. Goethe University, Frankfurt am Main, Germany; 7 Department of Internal Medicine III, Hematology and Oncology, University Hospital Regensburg, Regensburg, Germany; 8 Practice for Plastic and Aesthetic Surgery Munich, Munich, Germany; 9 Faculty of Law, University of Augsburg, Augsburg, Germany; 10 Department of Plastic, Aesthetic, Hand and Reconstructive Surgery, Hannover Medical School, Hannover, Germany; 11 Department of Hand, Plastic and Reconstructive Surgery, Microsurgery, Burn Center, BG Trauma Center Ludwigshafen, University of Heidelberg, Ludwigshafen, Germany; 12 Department of Surgery, Division of Plastic Surgery, Brigham and Womens Hospital, Harvard Medical School, Boston, USA

**Keywords:** Human Rights, European Convention on Human Rights, German Basic Law, European Law, Plastic Surgery, Reconstructive Surgery

## Abstract

**Abstract:**

Plastic surgeons are trained to perform a wide repertoire of surgeries—ranging from standard local procedures to highly specialized operations. Therefore, plastic surgeons treat a plethora of clinical presentations and address multiple patient needs. Their daily workflow is increasingly entwined with legal topics. The concrete legal interpretation falls within the remit of legal experts. However, by understanding the legal basics of selected surgical procedures, plastic surgeons may generate synergies in patient care and clinical practice. The legal situation is to be elucidated based on the German Basic Law (GBL) and the European Convention on Human Rights (ECHR).

**Level of Evidence V:**

"This journal requires that authors assign a level of evidence to each article. For a full description of these Evidence-Based Medicine ratings, please refer to the Table of Contents or the online Instructions to Authors www.springer.com/00266."

## Background

Plastic surgery rests on the four pillars of reconstructive, aesthetic, hand and burn surgery and has displayed steep growth over the past few decades. In 2019, the global plastic surgery workforce performed over 11.4 million cosmetic procedures [1]. One tenth of the entire workload was accumulated in the land of poets and thinkers. Thus, Germany is ranked among the top three countries with the most cosmetic operations worldwide. However, the magnitude of these numbers seems miniscule considering that approximately 30% of all diseases worldwide can be surgically treated and that more than 4.8 billion people are denied immediate surgical care [2, 3]. The plastic surgeons of today are willing to face this responsibility; both in humanitarian deployment and in everyday clinical practice [4–7]. They handle a wide range of clinical problems from cleft lip and palate to trauma and burn injuries [8, 9]. Jurisdiction gradually enters this field and creates legal principles, e.g., in preoperative outcome simulation, telemedicine, and digital patient-practitioner-communication [10–12]. However, legal aspects in plastic surgery are regularly misinterpreted to be a sword of Damocles and their purpose, to provide legal certainty for the patient and the physician, remains misunderstood [13–16]. However, a basic reciprocal understanding between law and medicine is paramount to uncover interdisciplinary synergies in patient care and clinical practice. Medical law by definition brings together both professions, but this profession requires specialized legal education, for example in the United Kingdom [17]. Hence, plastic surgeons have to be trained in basic legal thinking, which enables them to perform abstract analysis without shifting their scope from the scalpel.

While the medical providers speak a lingua franca that often allows for uncomplicated and direct knowledge transfer across borders, the jurisprudence features more individual principles that have to be interpreted in the context of the country-specific legislation and cannot be directly compared nor incorporated into different legal frameworks. Thus, we aimed to decipher the human rights constellation in an international legal system addressing over 500 million people—the European Convention on Human Rights (ECHR). To further depict national specifications, we deployed the legal framework of Europe’s largest economy—the German Basic Law (GBL).

Of note, the human rights-based approach pursued here is subject to several other competing rights and legal provisos. The reason is simply that fundamental rights are in principle not granted without limitations (e.g., Art. 8 para. 2 ECHR). In other words, patients' demands only have to be respected if they are within the applicable law of the respective national legal system. This applies, for example, if a potential patient requests an illegal procedure or if an examination reveals that the desired procedure does not meet the standard of medical care. In this case, patients' rights are principally not violated if surgeons refuse to comply with the request.

## Legal Synopsis

In principle, it is possible to invoke different human rights codifications from the German Basic Law (GBL) and the European Convention on Human Rights (ECHR). The determination of which particular human rights norm applies in an individual case begins with an examination of what type of protection is sought and whether a corresponding scope of protection is stipulated. Within the German legal system, the classification of human rights focusses on the Articles of the GBL, which directly refer to the human being. For this purpose, terms, such as "human being" (“*Mensch*”; e.g., Art. 1 para. 1 GBL), “everybody” ("*Jeder*"; e.g., Art. 2 para. 2 sentence 1 GBL), "person" (“*Person*”; e.g., Art. 2 para. 2 sentence 2 GBL), and “no one” ("*Niemand*"; e.g., Art. 3 para. 3 GBL) emphasize the distinctive reference to the human being. Further, civil rights accounting for a specific area of freedom, are defined as human rights (e.g., Art. 4 para. 1 GBL).

In contrast to the German Basic Law, the European Convention on Human Rights and its Section I ("Freedoms and Rights," Art. 2 - 18 ECHR) exclusively contain codifications that guarantee certain areas of freedom as human rights, irrespective of nationality. Subsequent to its universal wording, terms, such as "everyone" (e.g., in Art. 5 para. 1 sentence 1 ECHR), "no one" (e.g., in Art. 7 ECHR) characterize the ECHR, whereas "human" is only referenced as part of the term “human rights” and can be found in Art. 3 ECHR (“inhuman treatment”).In light of this pattern, the human rights of the ECHR and the fundamental rights of the GBL overlap, as shown in Table 1. The central pillars of the GBL and the ECHR often show the same structure, e.g., for the protection of life and physical integrity, and the freedom of the person. Although structural differences prevail, e.g., the human rights are based on the human being as such, the German Basic Law and the ECHR can rarely be applied simultaneously. By way of example, one should consider Art. 59 para. 2 sentence 1 GBL. This provision practically transforms the ECHR into so-called ordinary federal law. Following the hierarchy of norms, the system of superiority and inferiority of provisions, the ECHR is neither superior nor equal to the German Basic Law, but rather subordinate to it. Consequently, a complainant is not able to directly challenge the violation of a human right contained in the European Convention on Human Rights (ECHR) by a constitutional complaint before the German Federal Constitutional Court. This limitation finds its equivalent in the specific complaint procedure before the European Court of Human Rights (ECtHR) according to Art. 13 and 34 ECHR. Nevertheless, German courts must observe and apply the European Convention on Human Rights (ECHR) in interpreting national law. The guarantees of the Convention and its protocols, however, are not a direct constitutional basis for a court's review, if only because of the status given them by the German Basic Law (GBL). But on the level of constitutional law, the text of the Convention and the case-law of the European Court of Human Rights (ECtHR) serve as interpreting aids in determining the contents and scope of fundamental rights and fundamental constitutional principles of the German Basic Law (GBL), to the extent that this does not restrict or reduce the protection of the individual's fundamental rights under the GB.

**Table 1:** Summary of different legal asset points to textual congruences of the European Convention on Human Rights (ECHR) and Basic Law for the Federal Republic of Germany (GBL)

Basic law for the federal republic of germany (GBL)	European convention on human rights (ECHR)	Fundamentally protected area
Art. 1 Para. 1	Human rights safeguards, e.g., Art. 3 (prohibition of torture or inhuman or degrading treatment or punishment) and Art. 8 Para. 1 (right to respect for private and family life), and "spirit of the ECHR"	Human dignity
Art. 2 Para. 1 in conjunction with Art. 1 Para. 1	Art. 8 Para. 1, 1. Alt.	General right of personality
Art. 2 Para. 2 Sent. 1	Art. 2 Para. 1	Life, physical integrity
Art. 3 Para. 1	Art. 14 (only supplementary, no standalone meaning)	General principle of equality
Art. 3 Para. 2 Sent. 1	Art. 14 (only supplementary, no standalone meaning)	Equal rights irrespective of gender
Art. 4 Para. 1, 2	Art. 9 Para. 1	Freedom of religion
Art. 5 Para. 3 Sent. 1, 1st alt.	Art. 10 Para. 1 Sent, 1 and 2 (partially)	Artistic freedom

Despite the customized architecture of each legal framework, their commitment to humanism and reference to the individual human being is evident. Art, article; Para, para.; Sent, sentence.

## Human Right to Beauty

Situated at the crossroads between the German Basic Law (GBL) and the European Convention on Human Rights (ECHR), plastic surgery is associated with a plethora of legal challenges. The assumption of the right to beauty has been derived from the principle of human dignity [18]. Human dignity in the sense of the German Basic Law guarantees the claim of the human being to social value and respect and thus, in particular, the "protection of a narrower sphere of personal self-determination" and is the supreme constitutional value [19–22]. Thus, the guarantee of human dignity in Art. 1 para. 1 GBL and its absolute nature safeguards the human personality as such and the core areas of personal "individuality, identity and integrity." Consequently, some argue that this guarantee (as well as Art. 2 para. 1 GBL and Art. 8 ECHR) provides a solid basis for the patient's autonomy to determine his or her own health situation [23–25]. In contrast, the European Convention on Human Rights (ECHR) does not refer to the term "human dignity" explicitly, but the ECHR does emphasize the protection of human dignity as the "essence of the Convention" [26]. Furthermore, Art. 8 ECHR safeguards the right of every person to respect his or her private life as a distinct manifestation of human dignity. The concept of private life in this sense is broad and includes the physical and mental integrity, as well as the physical and social identity of the patient [27–29]. This guarantees the right—and thus the freedom—of the individual to make his or her own decisions about his or her body [30–32]. This right to self-determination also implies the right of the individual to decide for himself about the enhancement of his external aesthetic appeal—to this extent, there is a right to individual beauty. Nevertheless, in addition to this overarching legal context, the surgeon's practice depends on the actual and concrete legal implementation in everyday medical practice, for example at the level of criminal law—according to German judicature, surgery and cosmetic surgery in particular require the prior consent of the respective bearer of the fundamental right, which in turn requires the performance of appropriate informative duties on the part of the physician [33, 34]. Therefore, the acquisition of informed consent is, apart from the moral obligation, a necessity to adhere to the inviolability of a patient’s freedom and highlights the absence of obligation to undergo medical procedures. Accordingly, cosmetic procedures void of correctly informed consent by the patient depict a violation of the patient’s right to health and self-determination. Of note, this notion and its legal considerations apply even if the surgical procedure results in an improvement to the patient’s health. As for surgical procedures not legitimized by valid informed consent, surgeons could only rely on the connotation of necessity of treatment as a source of justification. However, this treatment validation is not applicable for aesthetic surgical procedures given that they are objectively not essential for preservation of health. This implies the necessity for meticulous risk-benefit analyses and consequently an obligation to provide detailed and thorough information prior to surgery. Note that a legally binding right to beauty may entail further implications, especially regarding insurance matters and extreme patient wishes.

Of note, cosmetic procedures are not covered by the statutory health insurance, for instance, on the German federal level—since they are medically not essential and necessary and performed solely for aesthetic reasons [35–37].

**Fig. 1:**
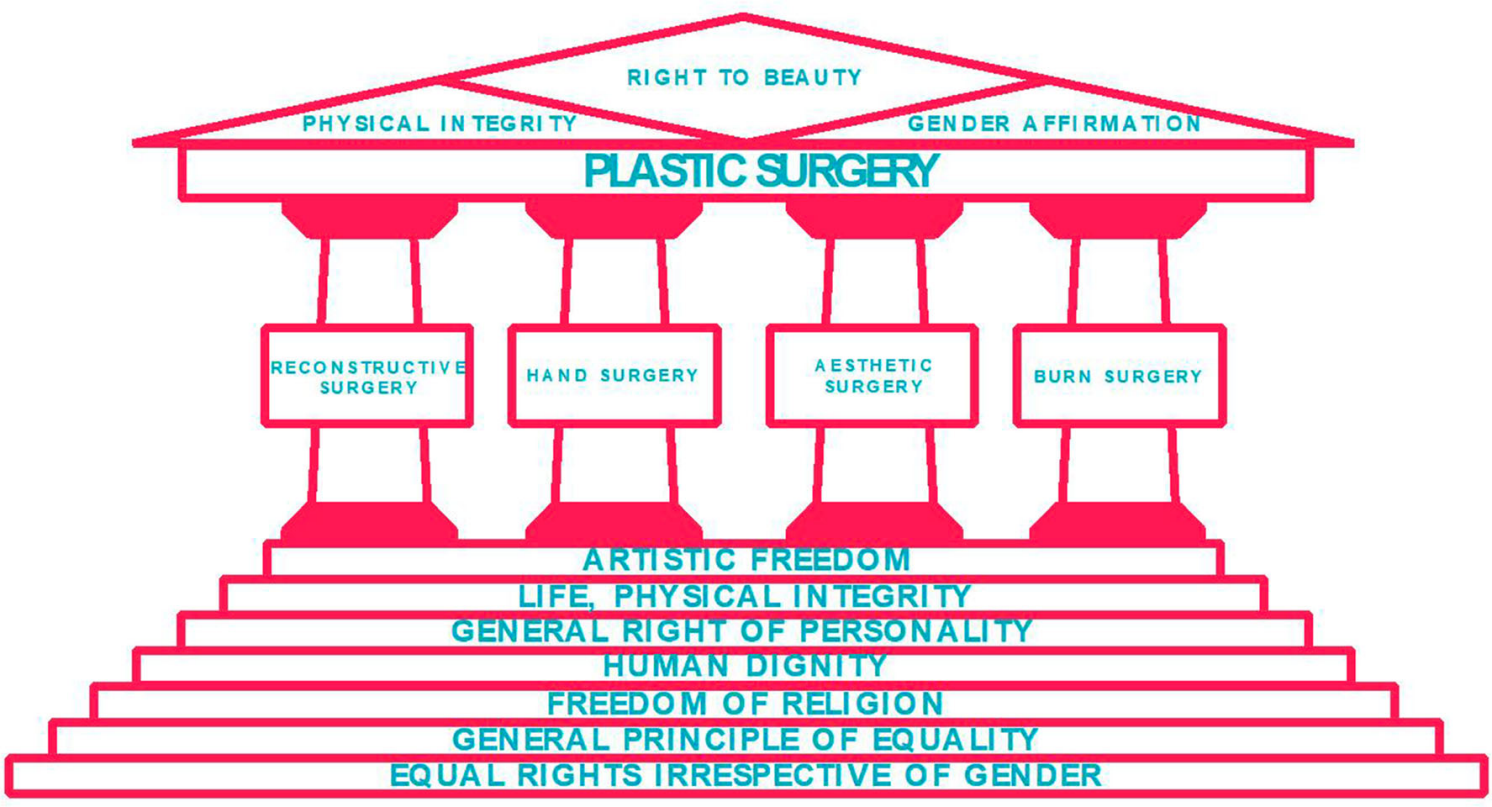
The pillars of plastic surgery and their organizational embedding into the jurisprudence. The overlapping legal principles between the European Convention on Human Rights (ECHR) and the German Basic Law (GBL) build the fundament for the four characteristic pillars of plastic surgery. The medico-legal synergy allows plastic surgeons to combine surgical skills and ethical as well as juristic aspects in addressing current debates and upcoming challenges in plastic surgery

The velocity of the uprising and diminishing of beauty trends must also be considered. The concept of "beauty" is in tune with the zeitgeist and remains constant only in its subjectivity and transformation [38, 39]. The pulse of these beauty trends is high-frequency, so that long-lasting and science-based beauty standards are difficult to establish [40]. Further, it needs clarification on what degree of intrusion into the life of the individual should be granted the right to beauty. It seems dystopian that representatives of plastic surgery, for example, proactively assess external shortcomings and take care of them, since this approach would entail the risk of selection; individuals would become patients against their will. This point of thought marks the watershed to radical convictions—in the operation room as well as in everyday life. This facet of medicine has been experienced by mankind, among others, in totalitarian regimes, and it was clearly rejected and discarded [41] (Fig. 1).

## Human Right to Physical Integrity

According to Art. 2 para. 2 sentence 1 GBL, each person has the right to physical integrity [42–44]. As such, both physical and mental health are protected. Furthermore, there is a corresponding right to self-determination of the individual—they decide on their physical and mental well-being [45–47]. This individual fundamental right is accompanied by a state duty to safeguard the physical integrity of the individual—"as a fundamental function of the state in general" [48–53]. The state must protect the individual against infringements of physical integrity and health [49, 54–56]. In the context of the European Convention on Human Rights (ECHR), in turn, Art. 8—protection of private life, with its relevant guarantees of freedom, is the decisive factor (see above); the provision safeguards and protects physical integrity at the level of European law. A corresponding duty of the state to protect is derived from Art. 2 para. 1 ECHR at the level of European law. Accordingly, the state must also ensure public health care [57–61]. In particular, the state shall ensure the adequate provision of care for patients in public and private hospitals [60, 62, 63]. Consequently, high professional standards must apply to the medical staff [60, 64]. These demanding requirements apply to the work of a plastic surgeon from the beginning of his residency. In Germany, plastic surgery residency requires six years of training, during which approximately 600 surgeries have to be performed independently. In addition, the specialist examination must be conducted by the respective state medical association [65]. This qualifies the plastic surgeon for reconstructive treatment of trauma and tumor patients, burn victims, and congenital defects, among others, and thus restores physical integrity [66–70]. The basic principles in reconstructive surgery with their increasingly complex therapeutic strategies for wound management are portrayed in the reconstructive ladder, which begins with healing by secondary intention and can extend to free flaps [71]. Also, there are specific time frames elaborated for different reconstructive procedures [72–74]. The defined target corridor of reconstructive interventions set concrete benchmarks. In these objectifiable frameworks, German jurisprudence finds clear points of leverage and can ensure legal certainty, such as in the dispute on the admissibility of liposuction by plastic surgeons, on the omission of reimbursement for reconstructive gender reassignment virilizing surgery or a non-medically indicated mammary augmentation, and on the insurance obligation for minimally invasive endoscopic reconstructive activity [75–78] (Fig. 1).

## The Human Right to Gender Equality

Considerable efforts for gender equality in plastic surgery are underway, both from the provider as well as patient viewpoint. In terms of surgeons, the percentage of women matriculated, female residents and representation in leading plastic surgery positions and editorial boards has been continuously increasing [79, 80]. To overcome the “plastic ceiling” referred to by Morain (1999), that is the omnipresent gender imparity in plastic surgery, the field of plastic surgery is endeavoring to further promote gender equality [81]. The Women Plastic Surgeons of Canada, the Women Plastic Surgeons Forum, and the association "*Die Chirurginnnen*" ("Women Surgeons") are exemplary results of these efforts [82]. Further, plastic surgery stands up for anti-discrimination campaigns targeting a wide array of inequities within this specialty [83].

## Gender Affirmation Surgery

Striving for gender equality in patient treatment, plastic surgery is on the forefront of gender affirmation surgery [84, 85]. Plastic surgery—together with other specialities—spearheads the medical care of patients' transition to their self-identified gender and addresses the discrepancy between 25 million patients suffering from gender dysphoria. A total of 660 gender affirmation surgeons exist in the US (status as of December 2020) while in Germany, surgeons perform over 2,000 gender affirmation surgeries per annum [86, 87]. With regard to the legal framework of gender reassignment surgery, the patient's right to self-determination continues to apply at the level of European and German federal law (see above); in this respect, the medical indication is decisive [88–90]. There are no other implications from the special fundamental right to equality under Art. 3 para. 2 sentence 1 GBL, according to which men and women have equal rights. This equality is not to be understood in the sense of "equality of sex," but with regard to equality before the law as such. In this context, only the general principle of equality according to Art. 3 para. 1 GBL, which states that all people are equal before the law, can gain importance. And here only insofar as it does not constitute a violation of this general principle of equality if individuals suffering from gender dysphoria under health insurance are denied comprehensive access to cosmetic surgery under health insurance law, which is denied to cis persons under health insurance from the very beginning [91, 92]. It is important that the patient's goals and expectations are precisely discussed, and possibilities and limits are outlined. Surgical gender affirmation should be explicitly desired by those affected. Individuals who define themselves as gender non-conform do not necessarily suffer from gender dysphoria and such do not seek gender affirming procedures [93]. A significant proportion of individuals suffering from gender dysphoria are perfectly happy with cross-gender hormonal treatment only, without seeking any surgical measures. Their constitutionally protected right to self-determination in the form of the right to find and recognize of one's own gender identity (Art. 2 para. 1 in conjunction with Art. 1 GBL as well as their right to bodily integrity (Art. 2 para. 2 sentence 1 GBL) is to be respected, of course. The same holds true for individuals who define themselves as non-binary. Needless to say, that no one should be forced into any conforming (surgical) measures (Fig. 1) [94].

## Outlook

The interface between elective aesthetic surgeries and the threat of medical liability is as complex as conceivably at any given time in history. The plastic surgeon’s exposure to professional liability remains exceptional regarding two aspects. Firstly, plastic surgeons performing elective cosmetic procedures do not contribute to the care of ill or traumatized patients to derive health*.* Rather the treatment’s goal aims to derive a healthy patient to better him or herself. Secondly, the surgical results will undergo judgment by the patient according to standards which are entirely personal and subjective.

With a focus on human rights at the level of European law and the German federal level, further important questions for plastic surgeons need to be clarified: For example, in cases of overlap; certain aesthetic interventions, such as genital circumcision, may also be covered by the human right to freedom of religion. Furthermore, body modifications, for example through subdermal implants, as an artistic expression have become more popular over the last decades. Therefore, one must consider whether aesthetic interventions could also be subject to artistic freedom, especially if the individual perceives the aesthetic changes to his or her body as a work of art. Future research work should elucidate the aforementioned concepts in comparison with the Anglo-American law to provide a transatlantic overview.

## Conclusion

The overlap between the GBL and ECHR highlights their humanitarian intention. Plastic surgery catalyzes the translation of human rights into public life. Therefore, we call on plastic surgeons to reevaluate their viewpoint on legal aspects in plastic surgery and exploit interdisciplinary synergies to further improve patient care and clinical practice.
